# Healthcare professionals’ perspective can guide post-marketing surveillance of artemisinin-based combination therapy in Uganda

**DOI:** 10.1186/s12936-020-3148-5

**Published:** 2020-02-10

**Authors:** Helen Byomire Ndagije, Ronald Kiguba, Leonard Manirakiza, Elijah Kirabira, Allan Sserwanga, Leah Nabirye, Jackson Mukonzo, Sten Olsson, Anne Spinewine, William D’Hoore, Niko Speybroeck

**Affiliations:** 1 National Pharmacovigilance Centre, National Drug Authority, Kampala, Uganda; 2 Department of Pharmacology and Therapeutics, Makerere University, Kampala, Uganda; 3 Pharmacovigilance Consulting, Uppsala, Sweden; 4 Institute of Health and Society (IRSS), Université catholique de Louvain, Brussels, Belgium

**Keywords:** Healthcare professional perspectives, Artemisinin-based combination therapy, Perceived treatment failure

## Abstract

**Background:**

Efficient testing to identify poor quality artemisinin-based combination therapy (ACT) is important to optimize efforts to control and eliminate malaria. Healthcare professionals interact with both ACT and malaria patients they treat and hence could observe, first-hand, suspect poor quality artemisinin-based combinations linked to poor malaria treatment outcomes and the factors associated with inappropriate use or treatment failure.

**Methods:**

A cross-sectional study of 685 HCP perspectives about the efficacy of ACT between June and July 2018 at selected health facilities in Uganda. Medicine samples were obtained from the seven regions of Uganda and tested for quality using the Germany Pharma Health Fund™ minilabs.

**Results:**

The average age of the 685 respondents was 30 (SD = 7.4) years. There was an almost equal distribution between male and female respondents (51:49), respectively. Seventy percent (n = 480) were diploma holders and the nurses contributed to half (49%, n = 334) of the study population. Sixty-one percent of the HCPs reported having ever encountered ACT failures while treating uncomplicated malaria. Nineteen percent of HCPs thought that dihydroartemisinin/piperaquine gave the most satisfactory patient treatment outcomes, while 80% HCPs thought that artemether/lumefantrine gave the least satisfactory patient treatment outcomes, possibly due to dosing schedule and pill burden. Healthcare professionals from the Central region (OR = 3.0, CI 0.3–1.0; P = 0.0001), Eastern region (OR = 5.4, CI 2.9–9.8; P = 0.0001) and Northern region (OR = 5.3, CI 2.9–9.9; P = 0.0001) had a higher chance of encountering ACT failure in 4 weeks prior to the survey as compared to those from the western region. Healthcare professionals from private health facilities also had higher chances of encountering ACT failures in past 4 weeks as compared to those from public health facilities (OR = 2.7, CI 1.7–3.9; P = 0.0001). All 192 samples passed the quality screening tests. The random sample of 10% of all samples randomly obtained by the laboratory staff also passed the chemical content analysis and dissolution tests.

**Conclusion:**

ACT medicines are widely available over-the-counter to the public and it is very difficult to report and monitor a decrease in efficacy or treatment failure. The perspectives of HCPs on treatment failure or lack of efficacy may potentially guide optimization efforts of sampling methodologies for the quality survey of ACT medicines.

## Background

The proliferation of substandard and falsified artemisinin-based combinations (SF ACT medicines) is of global concern and has disproportionately affected highly malaria-endemic Southeast Asian and sub-Sahara African (SSA) countries [1–4]. Up to 66% of drugs studied in SSA, in a recent quality study, were found to be substandard [5]. Newton et al. showed that SF ACT medicines account for 38% of artemisinin-based combinations in the private sector market in Uganda [6]. Studies have warned of a possible global spread of artemisinin-resistant parasites particularly to SSA [7].

The emergence of artemisinin-resistant strains of *Plasmodium falciparum* in SSA is of increasing concern [8] and likely results from decades of sub-therapeutic monotherapy and substandard artemisinin-derivative consumption [9]. Medicine quality is one of the many threats to appropriate and effective malaria case management and is critical in SSA countries using ACT as first-line therapy for malaria.

National Medicines Regulatory Authorities (NMRAs) in SSA can promote regular use of post-marketing surveillance (PMS) to curb the circulation of substandard, degraded, spurious, falsely labeled, falsified and counterfeit ACT medicines [10]. Post-marketing surveillance assesses the quality of ACT medicines on the market and stimulates the prompt identification and withdrawal of poor-quality medicines from public use. Uganda, a country with the sixth highest number of malaria-associated deaths in Africa annually [11], is among 21 of the 55 African countries with a PMS system [12].

Various sampling techniques, including convenience, mystery and overt approaches, have been used to estimate the scale of use of poor-quality ACTs in developing countries. Efficient testing to identify poor quality ACTs by use of resource-intensive high-performance liquid chromatography and mass spectrometry in developing countries [13] should focus on suspicious ACT brands. The three parallel supply chains for essential medicines in Uganda include the National Medical Stores for public health facilities, the public-not-for-profit supply chain led by the Joint Medical Sores and numerous procurement agents, manufacturers, distributors and retail pharmacies in the private-for-profit sector. Healthcare professionals interact with both ACT and malaria patients they treat and hence could observe, first-hand, suspect poor quality ACT medicines linked to poor malaria treatment outcomes [14]. A survey to establish treatment failure of ACT as perceived by HCPs could reveal invaluable insights into likely SF ACT medicines. These insights could guide PMS sampling of implicated ACT medicine brands by Uganda’s National Drug Authority. The implicated brands could be screened to evaluate their content of the active pharmaceutical ingredient and degradation products. Therefore, the objective of this study was to determine the extent of and factors associated with HCP-perceived ACT failure and identify the most implicated ACT medicine brands on the Ugandan market.

## Methods

### Study design

A cross-sectional study of healthcare professionals’ perspectives about the quality of ACT medicines between June and July 2018 at selected major hospitals and health centres, private community pharmacies and private not-for-profit clinics in seven regions of Uganda.

### Study setting

Health services delivery is decentralized within national, districts and health sub districts. The continuum of health services in the public sector begins with a village level community health extension worker, a parish level outpatient Health Centre II, a sub-county based 8-bed in-patient Health Centre III to a 12-bed Health Centre IV facility with a theatre manned by a medical doctor. In most districts, this network of facilities is complemented by a general hospital. The 14 regional referral hospitals are at the top of this continuum. The lowest level of care in the public health system is given by the community health workers who are volunteers in villages facilitating health promotion, service delivery, community participation, and empowerment. The private health sector in Uganda is diverse, comprising both public-not-for-profit organizations (faith-based, non-governmental, or community-based) and private-for-profit organizations (commercial, self-sustaining). The private sector players contribute to about 50% of the health service delivery [15].

The seven regions within which the National Drug Authority operates were included in this study to construct a nationally representative sample of health facilities in Uganda. One region is centrally located in Kampala and two regions distributed in each of the northern, eastern and western parts of the country. The central region is the most densely populated and is endowed with the majority of health facilities and drug outlets in the country. The West Nile and South West Regions both share borders with five neighboring countries. Each region was considered to be a cluster from which a random sample of health facilities would be drawn. The districts that were selected are Kampala (central), Iganga and Soroti (eastern), Arua and Lira (northern), Kabale and Fort Portal (western).

### Study population and sample size

In Uganda, an estimated 55,966 of the 66,111 people who make up the health workforce in 2009 were of the clinical cadre who are ideally eligible to participate in this study. To obtain the required sample size to be representative of the 55,9666 HCPs, the Krejcie and Morgan’s formula was used [16]. This gave a sample of 382 HCPs to be included in the study.

In order to cater for design effect and non-responses, the sample size was increased by the original eligibility proportion of 85% to get a sample size of 707 HCPs. During data collection, there was 3.1% (n = 22 HCPs) non-responses giving a total of 685 complete responses. The majority (42%) of these HCPs were in the central region; the northern had 17%, western 21%, and eastern 19%. The sample of 685 healthcare professionals included in the study achieved regional representation as follows: central (n = 295, 43%), northern (n = 132, 19%), western (n = 130, 19%) and eastern (n = 128, 19%).

Doctors, dentists and clinical officers (7837) represented 14% of the nationally eligible staff and were 35 of the overall sample. Pharmacists and pharmacy technicians (762) were 1.4% of the nationally eligible staff but 13% of our sample. Nurses, midwives, and nursing assistants (37,625) were 67% of the nationally eligible staff but 49% of our sample.

### Data collection procedure

The field team was trained on how to use the Open Data Kit (ODK) tools, field procedures and interview techniques. During the training, the android mobile phones of each field officer was configured and uploaded with the questionnaire in the ODK Collect tool. Additional file 1: File S1 shows more detail about the questionnaire that was used. This tool worked well with limited internet connectivity. Healthcare professionals in the selected health facilities completed the self-administered pre-formulated questionnaire. At the end of each day, each research assistant transferred the data from the paper questionnaire into the ODK tool then submitted the data to the central database server.

### Data analysis

The main outcome variable was the reported ACT failure or lack of efficacy. Treatment failure has been defined as the inability to clear parasites from a patient’s blood or to prevent their recrudescence after the administration of a drug [17]. This was in response to the quest for the HCPs observation or suspicion of any clinical failure to ACT. The HCP’s perception of ACT outcomes was used to identify the ACT medicine brands, registered in Uganda associated with perceived treatment outcomes to a pre-determined list of ACT medicines provided in the self-administered questionnaire. Perceived treatment failure was measured by the frequency with which a particular “brand” is listed. Factors associated with HCP-perceived ACT failure and adherence were explored including the demographic characteristics of the HCPs, their level of education and length of service as well as the type of health facilities where they worked.

Data were exported from the ODK tool database server into SPSS Version 20.0 for analysis. Binary logistic regression analysis was used to determine the factors associated with HCP-perceived ACT failure. Associations between independent variables (respondents’ gender, region, level of education, sector of practice, age, professional experience) and the outcome variable (HCP-perceived ACT failure) were assessed. Measures of association between categorical independent variables and the outcome variable were established using Chi-square tests. The measure of association for each categorical variable was the odds ratio (OR) with its 95% confidence intervals. The odds ratio was used to compare the chance of the outcome among the variables. Confounding and interaction between the various factors (region, gender, age, level of education, professional experience, sector of practice) were assessed using multivariate logistic regression.

### Quality tests for ACT medicines using minilabs

According to the World Health Organization (WHO) guidelines for selection of collection sites, [18], samples were collected, coded and recorded using the in a ratio sampling ratio of 1:3:1 representative of the 3 levels of distribution in the supply chain, respectively. Level one involved ports of entry, warehouses of the largest importers for both the public and private sector. The second level included wholesale pharmacies, retail pharmacies, drug shops, public and private hospitals, faith-based organizations, clinics and treatment centres and level three included informal outlets like kiosks, street vendors and grocery shops. Due to the complex nature of the environment at level 3 sites, covert sampling was used to obtain required samples.

Risk-based PMS sampling was performed after training and a risk estimation exercise for the regions of Uganda according to the United States Pharmacopoeial Convention guidance tool [19]. The choice of medicines was based on suspicion of or documented inferior quality with actual or potential serious implications for public health, such as treatment failures, high product turnover, exposure of patients to the medicine, seriousness of potential harm, products with a high potential for diversity on the market, where various generic alternatives are available as well as products at risk of being substandard and falsified due to potential high returns or demand in the market. Different brands of products containing artemisinin-based combinations were sampled from the seven National Drug Authority regional of Eastern, central, South Eastern, Northern, South Western, Western, Western and West Nile. The ACT medicines sampled included amodiaquine hydrochloride/artesunate tablets (AAT), artemether/lumefantrine tablets (ALT), artemether/lumefantrine dispersible tablets (ALD) and dihydro-artemisinin/piperaquine phosphate tablets (DPT). The sampling of ACT medicines was independent of the respondents and followed the Global Pharma Health Fund (GPHF) testing protocols [20]. The screening tests included visual inspection, disintegration test and qualitative analysis using the Thin Layer Chromatography (TLC) technique as prescribed in the GPHF-Minilab™. The dosage units, primary packaging, secondary packaging and product information insert were also inspected for any defects which were then recorded. A full monograph testing that assessed parameters of appearance, identification, chemical content and dissolution was performed in the National Drug Quality Control laboratory (NDQCL) on all failed, samples categorized as doubtful and 10% of all passing samples collected from each region.

## Results

### Demographic and professional characteristics of healthcare professionals

A total of 685 HCPs from the seven regions participated in the study with an average age of 30 (SD = 7.4) years. There were slightly more male (n = 349, 51%) than female (n = 336, 49%) respondents. The majority of HCPs had at least Diploma-level education (n = 480, 70%) with median experience of 3 (interquartile range, IQR of 2 to 6) years. Most of the HCPs were nurses (n = 334, 49%) and most participants were working in hospitals (n = 351, 51%). The majority of respondents worked in private facilities (n = 409, 60%). The median number of patients seen per day at the health facilities was 6 (IQR, 3 to 10) patients as summarized in Table 1.

**Table 1:** Demographic and professional characteristics of healthcare professionals

Total number of participants = 685	
Regions	
Central	295 (43%)
Eastern	128 (19%)
Northern	132 (19%)
Western	130 (19%)
Age	
Mean years (SD); median, IQR	30 (7.4); 28; 26–32
Gender	
Male	349 (51%)
Female	336 (49%)
Highest education level	
Certificate	205 (30%)
Diploma	245 (36%)
Bachelors’ degree or higher	235 (34%)
Professional cadre	
Pharmacist/Pharm tech	92 (13%)
Nurse	334 (49%)
Medical officer	110 (16%)
Clinical officer	114 (17%)
Others	35 (5%)
Professional experience (years)	
0–1 years	152 (22%)
2–3 years	217 (32%)
4–5 years	143 (21%)
≥ 6 years	173 (25%)
Health facility status	
Hospital or HC IV	354 (52%)
HC III/HC II	45 (7%)
Pharmacy/Pharm tech	123 (18%)
Drug shop	114 (17%)
Private clinic	163 (24%)
Sector of practice	
Public	276 (40%)
Private	409 (60%)

### Factors associated with having ever encountered ACT failure

Overall, a majority of the respondents reported having ever encountered ACT failure in treating uncomplicated malaria (n = 421, 61%) as shown in Table 2. Healthcare professionals from the central region had a higher chance of encountering ACT failure in 4 weeks prior to the survey as compared to those from the western region (OR = 3.0, CI 0.3–1.0; P = 0.0001). This was also true for the HCP from Eastern region (OR = 5.4, CI 2.9–9.8; P = 0.0001) and Northern region (OR = 5.3, CI 2.9–9.9; P = 0.0001).

**Table 2:** Extent of and factors associated with having ever encountered ACT treatment failure among 685 healthcare professionals using Multivariate logistic regression

Category	Ever encountered ACT treatment failure		OR	CI	P-value
	Yes, n (%)	No, n (%)			
Overall	421 (61)	264 (39)			
Region					
Western	50 (38)	80 (62)	1.0	–	–
Central	184 (62%)	111 (38%)	3.0	1.7–5.2	0.0001
Eastern	99 (77%)	29 (23%)	5.4	2.9–9.8	0.0001
Northern	88 (67%)	44 (33%)	5.3	2.9–9.9	0.0001
Gender					
Male	214 (61%)	135 (39%)	1.0	–	–
Female	207 (62%)	129 (38%)	0.8	0.6–1.2	0.311
Age category					
< 25	55 (49%)	47 (51%)	1.0	–	–
25–34	267 (61%)	172 (31%)	0.9	0.5–1.4	0.538
35 and above	99 (74%)	35 (26%)	1.1	0.5–2.5	0.734
Education level					
Certificate	129 (63%)	76 (37%)	1.0	–	–
Diploma	165 (67%)	80 (33%)	0.9	0.5–1.6	0.775
Bachelors or higher	127 (54%)	108 (46%)	1.0	0.5–1.9	0.973
Professional experience					
0–1 years	60 (39%)	92 (61%)	1.0	–	–
2–3 years	129 (59%)	88 (41)	1.2	1.6–5.6	0.511
4–5 years	104 (73%)	39 (27%)	2.4	2.2–10.2	0.003
≥ 6 years	128 (74%)	45 (26%)	3.2	1.6–11.4	0.001
Sector of practice					
Public	157 (57%)	119 (43%)	1.0	–	–
Private	264 (65%)	145 (35%)	2.7	1.7–3.9	0.0001
Professional cadre					
Medical officer	58 (53%)	52 (47%)	1.0	–	–
Pharmacist/Pharm tech	50 (54%)	42 (46%)	0.6	0.5–2.9	0.121
Nurse	218 (65%)	116 (35%)	0.7	1.3–7.2	0.337
Clinical officer	81 (71%)	33 (29%)	1.3	1.1–7.8	0.514
Other	14 (40%)	21 (60%)	0.2	0.5–4.5	0.110
Malaria patients per day					
< 10 patients	223 (52%)	215 (48%)	1.0	–	–
≥ 10 patients	188 (79%)	49 (21%)	2.5	1.6–6.6	0.0001

The longer the professional experience, the more likely a healthcare professional was to report an encounter of ACT failure in the previous 2 weeks. As compared to the health care professionals with experience of 1 year or less, those with experience of 4–5 years (OR = 2.4, CI 2.2–10.2; P = 0.0003) and 6 years and above (OR = 3.2, CI 1.6–11.4; P = 0.0001) had higher chances of encountering ACT failure in previous 2 weeks.

Healthcare professionals from private health facilities had higher chances of encountering ACT failures in the past 4 weeks as compared to those from public health facilities (OR = 2.7, CI 1.7–3.9; P = 0.0001). Healthcare professionals attending to more than 10 malaria patients a day had high chances of encountering ACT failure than those attending to less than 10 patients (OR = 2.5, CI 1.6–6.6; P = 0.0001).

### Commonly used artemisinin-based combinations

The study further investigated commonly used ACT medicines, suspected failures, and combinations that give satisfactory results in health facilities. The six most commonly used ACT medicine brands in the health facilities labelled and provided as Coartem, Lumartem, Duo-cotecxin, Lonart, P-Alaxin, Artefan. Coartem was the leading ACT drug suspected by health workers for treatment failure as illustrated in Additional file 2: Figure S1. The top five ACT failures associated drugs contributed to 80.2% of all suspicion and were all artemether/lumefantrine combinations).

### Drugs that were perceived to give satisfactory results in health facilities

Duo-cotecxin was ranked first with a score of 19.2% among the ACT medicines that gave satisfactory patient outcomes by health workers as demonstrated in an Additional file 2: Figure S2.

### Drug characteristics associated with perceived ACT failure

Some of the respondents agreed that ACT medicine colours (n = 183, 27%) could result in poor patient compliance, hence, treatment failure. Whereas some health workers explained that the colour of the medicine does not affect the effectiveness of the drug, they explained that some patients associated colour yellow with bitterness and, hence, nausea.

“*Most people are too much into political parties so some refuse to take yellow colored ACTs like Coartem*” Nurse, Northern region.

“*Patients tend to think yellow tablets are bitter probably due to past experience with bitter tablets that are yellow like chloroquine*” Nurse, Central region.

*“People are used to white tablets. They think yellow tablets are bitter. This causes poor compliance. The yellow colour together with the nauseating smell makes it hard to swallow such that patients end up vomiting*” Clinical officer, Eastern region.

Regarding the size of the ACT medicine tablets, some health workers (n = 285, 42%) agreed that it can lead to lack of compliance and hence treatment failure. They observed that artemether/lumefantrine and Duo-cotecxin tablets are relatively bigger and patients associated the size of Duo-cotecxin with ARVs. They explained this as follows:

*“Double strength Coartem tablets and Duo*-*cotecxin are bigger than most other ACTs. Despite the other advantages, they may be taken wrongly or halfway,”* Nurse, Eastern region.

*“If the size is big, it might be hard to ingest also swallowing many tablets at once induces nausea in some patients and hence vomiting out the medicines,”* Nurse, Western region.

*“Patients don’t like big tablets which resemble ARVs and that they may be perceived as HIV positive,”* Clinical officer, Northern region.

Most respondents agreed that the ACT drug taste (n = 470, 68%) could result in poor patient compliance, and eventually treatment failure. Health workers reported that most patients complain about the unpleasant taste of Coartem and makes them nauseous.

*“Bad taste affects the will to take the medication and sometimes result into nausea and vomiting,”* Clinical officer, Western region.

*“Children often resist swallowing them and also sometimes may lead to vomiting which discourages mothers from giving their children ACTs,”* Nurse, Central region.

Most of the respondents also agreed that dosing frequency (n = 411, 60%) and number of ACT medicine tablets (n = 562, 82%) could result in poor patient compliance, consequently treatment failure. Health workers explained that patients prefer drugs that are taken once a day such as Duo-cotecxin, as opposed to more than once daily. For example, patients do not usually finish doses of Coartem, Lumartem, Lonart, because they are twice daily dosing. They also pointed out that too many tablets of Coartem and Lumartem required per dose, bother patients and make it hard for them to complete doses. In their explanations they said:

*“Coartem, which has 4 tablets taken at once, for example, adult dose 4 tablets at once are many hence poor patient compliance”,* Nurse, Eastern region.

*“Coartem, you take 4 tabs morning and evening. It discourages patients. Once daily is preferred to 2 tablets twice daily. And if they have to co*-*administer with other drugs such as paracetamol, bill burden is high”,* Pharmacist, Northern region.

*“Coartem, Lonart, Lumartem oral tablets are too many. Patients don’t finish them”,* Nurse, Central region.

Inadequate knowledge about ACT drugs was another factor that was associated with poor patient compliance by majority health workers (n = 580, 85%). They explained that some health workers do not fully equip the patients with the information regarding drug use. They also explained that in Uganda, the literature about use of ACT medicines is limited.

*“ACTs have been in Uganda for sometime but literature on it is not enough. People still have to depend on the prescribers to determine use in pregnant mothers,”* Nurse, Northern Uganda.

*“Failure to warn a patient about an ACT’s potential side effects can lead to patients intentionally missing doses,”* Clinical Officer, Central region.

*“Some of the health workers don’t give patients full information on how to take the medicines,”* Nurse, Western region.

### Perceptions about ACT resistance

About one-third of health workers (32%, 220/685) felt that ACT resistance was a growing concern nationally. However, an equal number (31%, 210/685) felt otherwise. Thirty-seven percent of them (255/685) did not know whether drug resistance was of national importance.

More than a quarter of the health workers (28%, 192/685) felt that ACT resistance was a growing concern in their institutions whereas about one-fifth (22%, 148/685) felt otherwise. Half of them (50%, 345/685) did not know whether the ACT resistance was of concern in their institutions. Those who thought so associated the issue with patient self-medication and poor compliance as a great threat to ACT efficacy.

Respondents raised concern that malaria is still among the leading causes of hospital visits. The tendency for patients to self-medicate with over-the-counter ACT medicines and not complete the anti-malarial doses was considered to cause resistance especially if the anti-malarials were taken when malaria was not confirmed. They said:

*“ACTs are now our first line and when they fail we don’t have cheaper better alternatives so we need to be concerned. Also, the rate at which patients self*-*medicate is high”,* Nurse, Northern region.

*“A lot of patients do self*-*medication when they feel they have malaria symptoms even before confirming. Others abuse treatment given, they do not accomplish their doses, so after they start feeling a relief they stop”,* Clinical Officer, Eastern region.

*“ACTs are our first line for uncomplicated malaria and with cases of reports of resistance in South East Asia we need to worry that it may come to our nation”,* Medical Officer, Central region.

*“ACTs are widely used over the counter, this increases the chances of misuse by the patients and this can increase the risk of resistance”,* Nurse, Western region.

*“Because of easy uncontrolled access of these medicines to the general public and poor patient compliance whereby a patient takes one or two doses and stops when they get symptomatic relief”.* Clinical Officer, Northern region.

### The GPHF-Minilab™ test results

All samples passed the authenticity evaluation using the GPHF™ mini-lab. The sample distribution included 55% (106/192) samples of artemether/lumefantrine, 18% (35/192) of artesunate/amodiaquine and 27% (51/192) of dihydroartemisinin/piperaquine.

On visual inspection, 48 defects were detected, one-third (n = 16; 11 ALT, 1 AAT and 4 DPT) were dosage unit related. The commonest dosage unit related defects observed were orange peel and poor de-bossment. The orange peel defect is a coating-related defect that could be linked to the viscosity of the coating solution during manufacture. The four DPT defects were related to the tablet coating integrity and included orange peel, mottled tablets and dents on the tablet surface.

One quarter (n = 12) of the 48 reported defects were from covertly sampled artesunate/amodiaquine tablets in the West Nile region. These covert samples mainly from the informal sector had secondary packages with fading or dirty marks on the outside or inside of the carton and additionally, some had scratched blister packs. The samples appeared to have been exposed to poor sanitary conditions as some of them contained earth and dead insects in the secondary pack. Three of the AAT secondary packages were labelled as “Government of Uganda, Not for Sale”.

Twenty-three percent (n = 11; 5 ALT, 4 AAT and 2 ALD) of all observed defects were related to secondary packaging presenting mainly as varying degrees of black marks inside the cartons. The black marks were more common in cases where the carton contained more than 3 strips of dosage units.

There were seven (4 ALT, 3 AAT) primary package related defects that presented as scratches, creases or dirty marks on the primary packaging. Of concern also were two Lonart samples where the de-bossment of the expiry dates and batch number on the primary package were illegible and appeared to have been substituted with imprinting using ink. There were also two ALT cases with missing product information leaflets in all the collected samples.

All ACT samples that had been screened at the regions were transported to the National Drug Quality Control Laboratory for confirmatory testing. Since all samples obtained from the different regions had passed the screening stage, 10% of these samples were randomly selected and subjected to analysis following internal procedures. The tests conducted included appearance, identification, dissolution and assay for content of the active pharmaceutical ingredient. All the selected samples passed the full monograph second level testing as indicated in Table 3.

**Table 3:** Results for full monograph testing of the ACT drug samples from the NDA regions

Region	Samples sent for level II	Tested	Passed
Eastern	26	3	3
Central	24	2	2
South Eastern	33	3	3
Northern	26	3	3
South Western	23	2	2
Western	31	3	3
West Nile	29	3	3
Total	192	19	19

## Discussion

In this study, six out of every ten healthcare professionals perceived having encountered treatment failure while treating uncomplicated malaria. The highest level of perceived treatment failure was reported among the artemether–lumefantrine (AL) containing ACT medicine brands of Coartem, Lumartem, Artefan and Lonart. On further probing, one of the factors likely to be associated with the HCP perceived treatment failure was the region of work. This perceived treatment failure was validated with a countrywide regional sampling and testing of oral ACT medicines. Some product defects were detected on visual inspection. The West Nile Region stood out with one quarter of the visual defects because sampling was done covertly from the informal sector in this region on the border with the Democratic Republic of Congo and Southern Sudan. However, from the laboratory analysis, the quality of anti-malarial medicines in all the regions of Uganda was found to be good. Regulatory quality assurance measures such as post-market surveillance contribute considerably to prevention, detection and response to the circulation of substandard medicines on the market but are resource intensive [21]. Insights from healthcare professionals who interact much with the patients and with the medicines result in more targeted and cost-effective PMS efforts especially for limited resource settings.

Healthcare professionals are one of the important partners in tackling the problem of substandard and falsified medical products on the market [1]. Their perspectives count especially in detecting and preventing the circulation of these products. National medicines regulatory authorities can take cues from them during PMS. In this study, insights from HCPs guided the covert sampling that led to the discovery of visually defective products in the informal sector that could have easily crossed the border into neighbouring countries. Thus, at least three things need to be investigated further even though they did not affect the chemical content analysis of the concerned products at our internationally recognized NDQCL [22]. Earlier studies have reported presence of non-quality assured anti-malarial medicines in many sub-Saharan countries including Uganda [23, 24]. Firstly, possible pilferage of government drugs with the cooperation of other stakeholders like the local government authorities, customs officials, the police and other law enforcement agencies in the affected region. The drug regulatory authority also needs to conduct more covert operations more regularly in similarly affected regions. This very resource-intensive exercise is not easily sustained by many low and middle-income countries but can be better implemented by cross-border regional collaboration. Modification of the WHO methodology can also be made to conserve medicine samples and also include them among those that can contribute to the next stage sampling if they can be visually inspected without damaging their primary packaging. Secondly, coating defects for ALT and DPT could indicate a possible break down during the manufacturing process or possible falsification. The respective manufacturers need to be notified about this. Lastly, the cases of missing product information leaflets (PIL) of all samples of a drug that were collected needs to be addressed as soon as possible and the regulatory requirement for PILs should be enforced strictly as it could also be an indicator of product falsification.

From the perceptions of healthcare professionals in this study, the once-a-day dosing schedule and low pill burden of dihydroartemisinin/piperaquine (DHP) was considered to be more favourable to patients and to have more satisfactory treatment outcomes than the AL-containing brands. This finding was similar to another study that recommended the use of DHP in the Ugandan malaria treatment policy due to its less intense dosing schedule and requirements [25]. The top four most frequently used AL brands of Coartem, Lumartem, Artefan and Lonart suspected to be failing to result in the desired patient outcome in this study contributed to 80% of the suspicion. Misdiagnosis of non-malaria febrile illness as malaria and self-treatment with anti-malarials, especially in areas with limited access to health services [26, 27] has greatly contributed to the overuse of the AL containing ACT medicine brands and possibly to the notion among HCPs that this group of medicines do not give the desired outcomes. A bioequivalence analysis of these four brands with the highest perceived treatment failure rates is highly recommended to verify this view. In addition, the efficacy of 5-day and 7-day ACT regimens against the standard 3-day regimen in treatment of uncomplicated malaria would also need to be evaluated to support the malaria policy. As a follow on for policy makers, mapping and predicting ACT resistance trends would then provide information necessary to take early action to preserve the performance of these drugs for as long as possible, also taking into account the resources needed in a good prediction model. The relationship between ACT resistance and ACT use in the various regions also needs to be established in future studies. The outcome of such studies would be of much interest to the drug regulators since bioequivalence analysis is not a pre-condition for registration of medicines, yet it determines the performance of the many generics that are used on the Ugandan market. The test, treat and track malaria policy in Uganda [28] if reinforced will be beneficial in terms of reducing the cost of treatment and quality of healthcare provided.

It was found that the longer one’s professional experience, the more likely it was to acknowledge the encounter of perceived treatment failure. One study has shown that although an individual health worker’s years of experience influence their level of expertise, gains in the probability of an individual worker being an expert can also be achieved through having more years of service and more educated staff overall [29, 30]. The hospital practice context and the general work environment greatly enhances the clinical practice expertise, including reporting and handling treatment failure in the context of this study. Thus, in the search for better health worker performance, other factors such as job satisfaction, workload and other health system factors may be important considerations [31]. These factors will need a deeper understanding for the NPC to establish and maintain an effective system for managing treatment failure or lack of efficacy. The accreditation of the NPC as a provider of continuing professional education points will also increase the level of pharmacovigilance awareness and engagement among the different medical professions through their professional associations.

Poor adherence to medicines and drug resistance were identified, in this study, as the main factors that could lead to possible perceived treatment failure. In general, HCPs agreed that dosing schedules, pill burden and drug aesthetics affect patient adherence [32, 33]. Since the majority of respondents spent the most time with patients, they described the patients’ views in detail. However, future studies could get more accurate patients’ views directly from the patients. A study in Ghana reported that 57.3% of patients were adherent to prescribed ACTs [24]. About one quarter of the HCPs associated yellow colour with the political party in power and also with bitterness, making the AL brands unpopular in some regions. The ACTs tend to be bigger than the older anti-malarial medicines. Despite the once daily dosing schedule that dihydroartemisinin/piperaquine was favoured for, the big size of these tablets likened it to anti-retroviral medicines, which are associated with the stigma of HIV in the community. Majority of the HCPs identified the bad taste of the artemether/lumefantrine brands to affect the will of the patients to take medicines. When the prejudice of patients and healthcare professionals are sufficiently handled with proper education at the point of care, adherence to prescription medicines will most likely improve. The views of patients and consumers have been used in high-resourced countries to report ineffective drugs through a labour intensive manual process [34]. However, this manual process of reporting drug quality problems could be adapted for low and middle-income countries like Uganda due to a higher prevalence of substandard and falsified products on the market.

The majority of health workers, interviewed for this study, neither thought nor knew that ACT resistance was a growing concern nationally. The WHO states that ACT resistance is growing worldwide. Non-synonymous propeller-region *PfKelch 13* mutants associated with delayed clearance have been reported in Africa [17]. What may be of greater concern still, is the presence of multicopy Pfplasmepsin 2–3 that could lead to piperaquine resistance [35]. In Uganda, *Plasmodium* genotypes with decreased sensitivity to artemether–lumefantrine increased from 2008 to 2012 [36]. Elsewhere, there are increasing reports of the emergence of less sensitive strains of *Plasmodium* to artemisinin, lumefantrine and piperaquine [37, 38]. The issue raised here is the sources of health literacy for the majority of HCPs are limited. However, the smartphone coverage for them could be explored with the intention of introducing a smartphone application with personalized medicine information. For the national malaria programme and NPC, routine monitoring for emergence of artemisinin resistance to ensure that the recommended ACT medicines are effective for as long as possible and that timely changes to national treatment policies can be implemented. Many health workers think that the threat to ACT efficacy is mainly from self-medication and poor compliance to anti-malarial treatment. Self-medication is common in Uganda [39]. However, lack of awareness of the regulations regarding the appropriate use of medicines, illiteracy and poverty have been cited as major drivers to self-medication. Therefore, major communication and education efforts have to be customized to the general public as well as HCPs.

The major limitation of this study was the use of healthcare professionals’ perspectives as a surrogate measure to assess lack of efficacy or treatment failure. Healthcare professionals do not usually report this. For all cases that mentioned having ever encountered ACT treatment failure, a confirmatory visit was made to the health facility to rule out brand confusion. A prospective observational design would have given better evidence about the treatment failure. However, since this outcome is very rare, such a study was esteemed to be too resource intensive in terms of time and funds. Like other cross-sectional surveys, biases in the survey instrument, information bias, interviewer, recall and selection bias could have affected the internal validity of the study. Since ACT failure was a new concept in adverse event reporting, a 4-week period was used instead of 2 weeks so as to enable capture of more encounters with the outcome variable. Selection bias was of greater concern due to sampling of both the HCPs and the ACT medicines. Health facilities in the regions were sampled in consideration of the national ratios as much as possible. The sampling of ACT was independent of the respondents and followed the GPHF testing protocols and the WHO guidelines for collecting samples for quality surveys of medicines. The national estimates for HCPs were more than 10 years old and could have also affected the representativeness of our samples. Lastly, the results may be generalized with caution to other similar low-and-middle income countries to the Ugandan health system context. Given the low number of valid samples in some categories of ACT due to convenience risk-based sampling, generalization of the observations may not hold statistical validity and a targeted PMS strategy should be adopted to give a more in depth and generalizable assessment.

The findings here can serve as a basis for the search into more cost-effective means to identify leads for ACT optimization and PMS sampling methodologies in sub-Saharan Africa. Sub-Saharan countries are net importers of pharmaceutical products and have recently individually or regionally began to build or boost their PMS and pharmacovigilance systems. HCPs have given their views; however, future studies should concentrate on acquiring patients’ views on the performance of medicines directly. Some ACT medicine brands were identified by HCPs. It would be very useful to conduct bioequivalence studies to verify treatment failure and to follow up on identified cues to product falsification or pilferage of medicines with the relevant authorities. Continued resistance monitoring to identify early artemisinin resistance and re-inforcement of the “diagnosis before treatment” policies would be of great value to national malaria treatment programmes. For the HCPs, it would be a worthwhile venture to explore the best means of availing them personalized health and drug information to aid their decision making on a real-time basis.

## Conclusion

Currently, malaria endemic African countries use ACT for treating uncomplicated malaria. The ACT medicines are widely available over-the-counter to the public and it is very difficult to report and monitor a decrease in efficacy or treatment failure. Clinical efficacy studies for the combination ACT medicines or any sort of post-marketing quality control tests are very resource-intensive and complex. Findings from this study will inform policy of efficient means of screening for substandard and falsified products on the market in Uganda and other low-income countries. The perception of most of the healthcare professionals interviewed in this study was that the risk of ACT failure has been associated with both their overuse that is commonly associated with high rates of re-infection and substandard drug formulations. In settings with limited resources, a risk-based approach is necessary to select mainly high-risk brands for testing. Since HCPs, during patient care, always interact with medicines, their opinions made on the basis of observing the ways in which patients respond to medicines can guide their selection of high-risk brands in order to carry out sustainable quality testing. However, other quality assurance measures should be maintained while the search for more cost-effective identification of leads for PMS investigation continues.

In addition, other behavioural factors, which could lead to perceived treatment failure and antimalarial resistance identified in this study need to be addressed. Of importance is poor patient adherence, which could be improved if patients are properly educated in medicine use at the point of care. Proper education for patients needs to address their concerns on colour, size, and number of tablets prescribed based on patient-based survey results. Healthcare professionals should also be empowered with information to make more informed decisions at the point of care. Future studies helpful in dealing with ACT resistance and ACT failure will benefit from simulation over a long period to come up with cost-effective strategies aimed at conserving efficacy of artemisinin-based combinations.

## Supplementary information

**Additional file 1: File S1.** Study questionnaire to assess clinician-perceived failure rates of commonly used ACT medicines. This was the questionnaire used to collect data about the HCP perceived failure rates of commonly used ACTs. Healthcare professionals in the selected health facilities completed this self-administered formulated questionnaire.

**Additional file 2: Figure S1.** Profile of suspected ACT failure rates in health facilities. A graphical representation of the percentages of drugs that were perceived not to have had satisfactory patient outcomes.

**Additional file 3: Figure S2.** ACT drugs with perceived satisfactory results.pdf. A representation of the percentages of drugs that were perceived to have had satisfactory patient outcomes.

## Data Availability

The datasets generated and analysed during the current study are not publicly available but are available from the corresponding author on reasonable request.
