# Supplementary material for: Healthcare professionals’ perspective can guide post-marketing surveillance of artemisinin-based combination therapy in Uganda
Source: Malar J. 2020 Feb 10;19:63. doi: 10.1186/s12936-020-3148-5 (PMC7011371; doi:10.1186/s12936-020-3148-5)
Supplement: Supplementary file 1 — Additional file 1: File S1. Study questionnaire to assess clinician-perceived failure rates of commonly used ACT medicines. This was the questionnaire used to collect data about the HCP perceived failure rates of commonly used ACTs. Healthcare professionals in the selected health facilities completed this self-administered formulated questionnaire. [file 12936_2020_3148_MOESM1_ESM.pdf]

A study to assess Clinician-perceived failure rates of commonly used ACTs

Interviewer's Name: ____________________ Code: [ ]

Region: ______________________________ Code: [ ]

Health Facility Name: ____________________________ Code: [ ]

Date of Interview: ____/_____/_____

Clinician-perceived ACT Treatment Failure: *The failure of a malaria-patient to improve despite having received an Artemisinin Combination Therapy (ACT) regardless of artemisinin resistance.*

SECTION A: DEMOGRAPHICS

1. Gender: [1] Male [2] Female

2. Age (*in completed years*): ………………….

3. Education Level:
   [1] Certificate
   [2] Diploma
   [3] Bachelor
   [4] Masters
   [5] Other (*specify*).______________

4. Professional experience (Years): [ ]

5. If less than 1 year in **Q4**, state number of completed months ……

6. Professional Cadre:
   [1] Physician
   [2] Medical Officer
   [3] Pharmacist
   [4] Nurse
   [5] Clinical Officer
   [6] Pharmacy Technician
   [7] Other (*specify*).______________

7. Health Facility Type:
   [1] Public
   [2] Private Not-for-Profit
   [3] Private for-Profit

8. Health Facility Status:
   [1] Hospital
   [2] Health Centre III
   [3] Health Centre II
   [4] Private Clinic
   [5] Pharmacy
   [6] Drug Shop
   [7] Other, (*specify*).______________

SECTION B: PERCEIVED ACT FAILURE

Please, complete the questionnaire by indicating the appropriate responses.

1. What is the approximate number of malaria-patients you see per day? ……………

2. In the use of ACTs in treating uncomplicated malaria, have you ever encountered any treatment failure(s) in your malaria-patients?
   [1] Yes [2] No

3. Have you suspected any ACT treatment failure in the past 4 weeks?
   [1] Yes [2] No

4. If ***YES*** to **Q3**, how many cases of ACT treatment failure? ....

5. Have you received patient-complaints of ACT treatment failure in the past 4 weeks? [1] Yes [2] No

6. If ***YES*** to **Q5**, how many patient-complaints of ACT treatment failure? …………

7. Briefly describe the most recent case of ACT treatment failure you have encountered providing information on patient age, brand of ACT involved, clinical outcome & action taken; e.t.c.

___

___

___

8. Have you reported any ACT treatment failure(s) in the past 6-months? (***Please tick one***)
   [1] Yes [2] No (***Skip to 13***)

9. If ***YES*** to **Q8**, to whom have you reported the most recent ACT treatment failure(s)? (***Please tick all applicable***)
   [1] District Health officer
   [2] Health Management Information System
   [3] Immediate Supervisor
   [4] National Pharmacovigilance Center
   [5] Others, (*specify*). ___________________

10. If ***YES*** to **Q8**, how did you report the most recent ACT treatment failure(s)? (***Please tick all applicable***)
    [1] Verbally
    [2] Written report
    [3] Other, (*specify*)…………………………………………….

11. What motivates you to report ACT treatment failure(s)?

____

____

____

12. Do you get feedback on the ACT treatment failure(s) you report?
[1] Yes [2] No

13. Do you feel that circumstances in your setting make it difficult to report treatment failure to ACTs? [1] Yes [2] No

14. Explain your response to **Q13**

____

____

____

15. What can be done to improve the reporting of treatment failure to ACTs in your setting?

____

____

____

16. What are the commonly used ACTs at your health facility? (***Please tick all appropriate***)

**Brand**			Coartem	
D-Artepp (GPSC)			Lumartem	
Artequin			Malfan	
Combiart			Artem	
Ridmal			Arexel	
Glumac			Lonart	
Duocotecxin			Lumether	
P-Alaxin			Lumaren	
Artefan			Other, (*specify*)………………………	

17. Which ACT brand(s) have you observed to result in treatment failure? (***Please tick all appropriate***)

**Brand**			Coartem	
D-Artepp (GPSC)			Lumartem	
Artequin			Malfan	
Combiart			Artem	
Ridmal			Arexel	
Glumac			Lonart	
Duocotecxin			Lumether	
P-Alaxin			Lumaren	
Artefan			Other, (*specify*) ………………………	

18. Do you think ACT resistance is a growing concern nationally?
[1] Yes [2] No [9] Don't Know

19. If yes to **Q18,** briefly describe why?

____
____
____

20. Do you think ACT resistance is a growing concern in your institution?
[1] Yes [2] No [9] Don't Know

21. If yes to **Q20**, briefly describe why?

____
____
____

SECTION C: DRUG FACTORS RELATED TO ACT FAILURE

22. Do you think the **color** of an ACT could lead to poor patient compliance hence treatment failure? [1] Yes [2] No

23. Briefly describe why giving examples?

____
____
____

24. Do you think the **taste** of an ACT could lead to poor patient compliance hence treatment failure? [1] Yes [2] No

25. Briefly describe why giving examples?

____
____
____

26. Do you think the **size** of an ACT tablets could lead to poor patient compliance hence treatment failure? [1] Yes [2] No

27. Briefly describe why giving examples?

____
____
____

28. Do you think the number of tablets swallowed could lead to poor patient compliance hence treatment failure? [1] Yes [2] No

29. Briefly describe why giving examples?

____
____
____

30. Do you think that inadequate information about an ACT could lead to patient misuse of the drug hence treatment failure? [1] Yes [2] No

31. Briefly describe why giving examples?

____
____
____

32. Do you think the dosing frequency of an ACT could lead to poor patient compliance hence treatment failure? [1] Yes [2] No

33. Briefly describe why giving examples?

____
____
____

34. What other factors in the practice of <u>patients</u> are responsible for the poor response of patients to ACT? (***Please briefly outline***)

____
____
____
____

35. What other factors in the practice of <u>clinicians</u> are responsible for the poor response of patients to ACT? (***Please briefly outline***)

____
____
____
____

We appreciate your time taken to respond to this questionnaire. Thank you

This a collaborative study between
National Drug Authority and Makerere University Department of Pharmacology and therapeutics.
