# Supplementary figures and images for: Healthcare professionals’ perspective can guide post-marketing surveillance of artemisinin-based combination therapy in Uganda
Source: Malar J. 2020 Feb 10;19:63. doi: 10.1186/s12936-020-3148-5 (PMC7011371; doi:10.1186/s12936-020-3148-5)

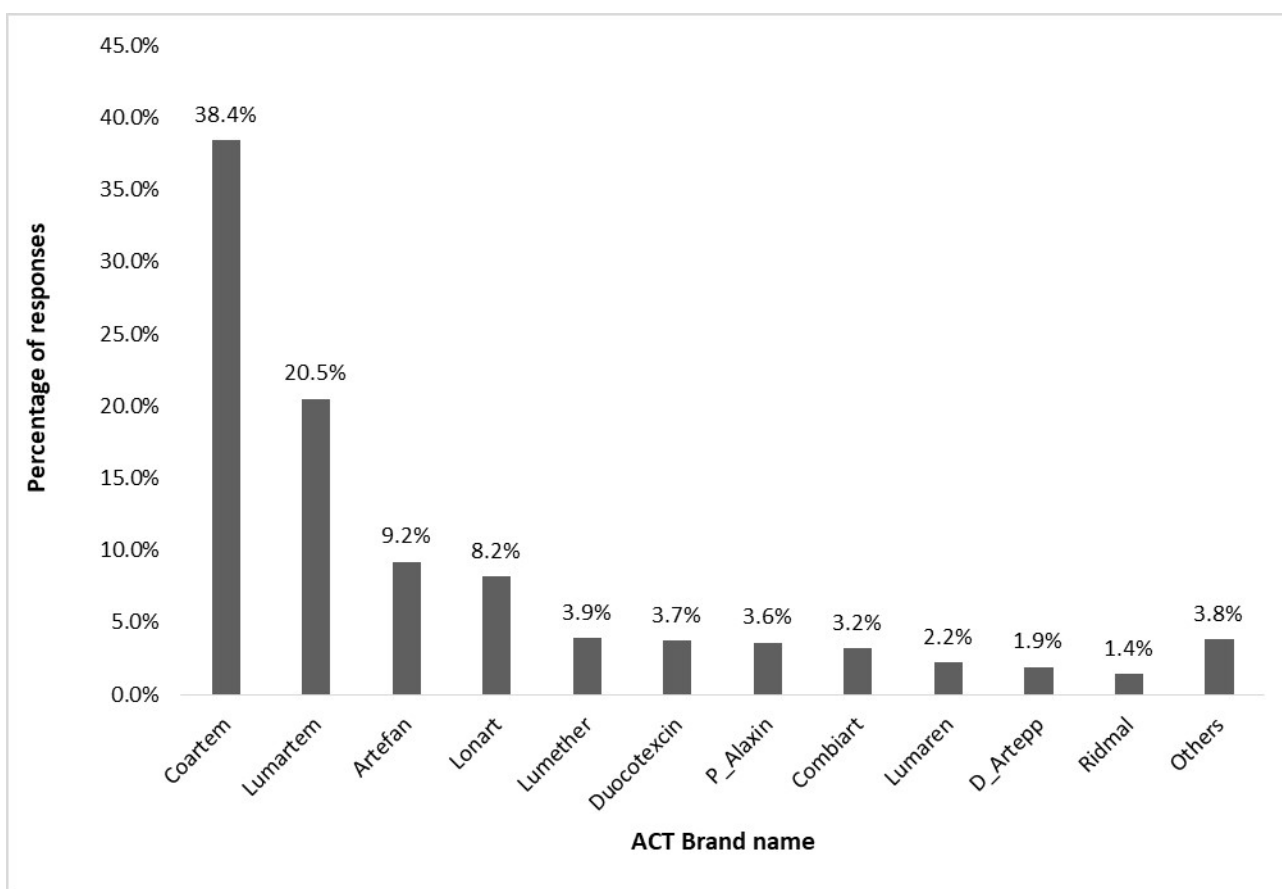

Supplement: Supplementary file 2 — Additional file 2: Figure S1. Profile of suspected ACT failure rates in health facilities. A graphical representation of the percentages of drugs that were perceived not to have had satisfactory patient outcomes. [file 12936_2020_3148_MOESM2_ESM.pdf]

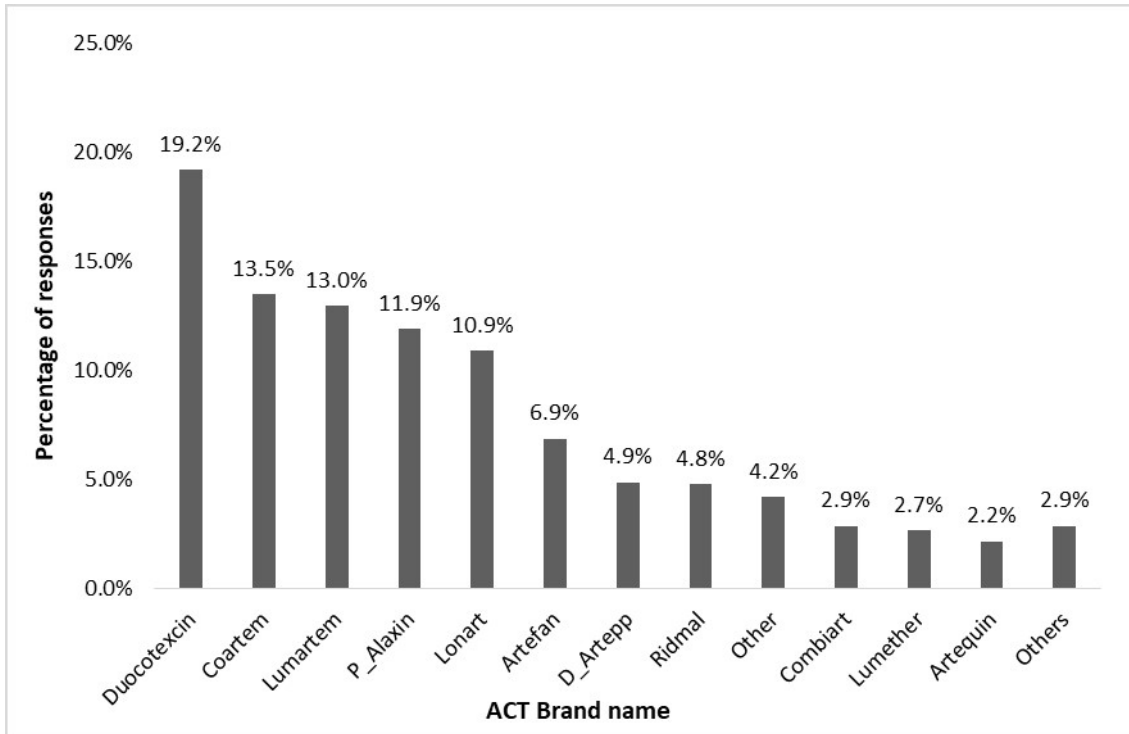

Supplement: Supplementary file 3 — Additional file 3: Figure S2. ACT drugs with perceived satisfactory results.pdf. A representation of the percentages of drugs that were perceived to have had satisfactory patient outcomes. [file 12936_2020_3148_MOESM3_ESM.pdf]
